# Facilitators and barriers to help-seeking for breast and cervical cancer symptoms: a qualitative study with an ethnically diverse sample in London

**DOI:** 10.1002/pon.3464

**Published:** 2013-12-19

**Authors:** Laura A V Marlow, Lesley M McGregor, James Y Nazroo, Jane Wardle

**Affiliations:** 1 Cancer Research UK Health Behaviour Research Centre, Department of Epidemiology and Public Health, UCLGower Street, London, UK; 2 CCSR, School of Social Sciences, University of ManchesterManchester, UK

## Abstract

**Objective:**

Earlier diagnosis of cancer has become a policy priority. There is evidence that minority ethnic groups are more likely to delay help-seeking for cancer symptoms, but few studies have explored reasons for delay in these groups. The present study explored facilitators and barriers to help-seeking for breast and cervical cancer in an ethnically diverse sample of women.

**Methods:**

Semi-structured interviews were carried out with 54 healthy women from a range of ethnic backgrounds; Indian, Pakistani, Bangladeshi, Caribbean, African, Black British, Black other, White British and White other. Framework analysis was used to identify themes.

**Results:**

Appraising a symptom as possibly due to cancer was an important facilitator of help-seeking, although for some the prospect of a cancer diagnosis was a deterrent. Women believed that earlier diagnosis improved the chance of survival, and this facilitated prompt help-seeking. A sympathetic GP facilitated help-seeking, and an unsympathetic GP was a deterrent. Some ethnic minority women described the use of alternative medicine and prayer as a first-line strategy that might delay help-seeking. Language barriers, racism and a tendency to ‘soldier on’ were also mentioned by these women.

**Conclusions:**

Models of delay in presentation for early cancer symptoms are likely to transfer across different ethnic groups. Encouraging open discussion about cancer among minority communities could help raise awareness about the importance of early detection and promote help-seeking as a priority response to a possible cancer symptom. © 2013 The Authors. *Psycho-Oncology* published by John Wiley & Sons, Ltd.

## Introduction

Presentation to a health professional with a cancer symptom begins the process of diagnosis and treatment. This will usually be preceded by a variable time between first recognising the symptom and seeking help; sometimes referred to as patient delay 1. Delays of more than 3 months are associated with a more advanced stage at diagnosis for some cancers 2–4, and diagnosis of more advanced disease is associated with poorer prognosis 5,6. Although most of the evidence supporting early diagnosis for improved outcomes has been in breast cancer, earlier diagnosis of all cancers has recently become a priority in policy 7.

Black, Asian and Minority Ethnic (BAME) groups are more likely to delay help-seeking 8–10, and as a result present at a more advanced stage for some cancers 11–14. In 2009, a survey of BAME men and women in the UK showed comparatively lower rates of anticipated help-seeking for most cancer symptoms than a nationally-representative survey carried out at the same time 15,16. Other demographic factors associated with delayed help-seeking include older age and being male, whereas findings regarding socio-economic status are unclear 9.

A range of factors are thought to be associated with delayed help-seeking. For example, more intrusive symptoms such as pain and bleeding result in shorter delays, as do symptoms that restrict daily activities 17. Psychosocial factors such as symptom awareness or attribution, fear and social support have also been implicated 8,9,17,18. The majority of quantitative studies contributing to this literature have been in ethnically homogenous samples, making it difficult to determine whether findings would be generalisable to diverse populations. In London, for example, 2011 census data indicates that the BAME population constitutes 40% of the total population of 8 million 19. For practitioners working in such ethnically diverse locations, research in appropriate populations is vital to ensure evidence-based decisions can address policy goals.

Recent surveys which have asked participants to select barriers to presenting with a cancer symptom from a predefined list, suggest the most common among BAME groups are worry about what the doctor might find, difficulty making an appointment and competing priorities 15. South Asian women seem more likely to report emotional barriers (e.g. fear and embarrassment) than White women when focussing on breast cancer 20. However, offering prompted lists of barriers makes it difficult to ascertain whether additional barriers would have been cited in response to an open question, or to explore barriers further in relation to their origin. In addition, theoretical models of symptom delay emphasise the importance of considering facilitators to help-seeking as well as barriers 1. At the time of this study to our knowledge, there were no published qualitative investigations of delayed help-seeking among ethnic minorities in the UK. So this study was designed to explore the facilitators and barriers to presenting to a health care professional with a possible symptom of breast or cervical cancer by using in-depth interviews. The aim of the study was to explore if and how facilitators or barriers to help-seeking discussed by BAME women differ from those identified by White British women.

## Methods

### Participants

A purposive sampling frame was devised with the aim of recruiting 45 women from Black Caribbean, Indian and a comparison group from White backgrounds (15 from each background spread across ages 25–64 years). When determining the sample size, we took into consideration the scope of the study, nature of the topic, study design and selection criteria 21,22 as well as the sample size of similar studies. Black Caribbean and Indian women were selected because they represent two of the largest UK BAME groups and are likely to speak English.^1^ The inclusion criteria were adapted once interviewing began for several reasons (i) identification of ethnicity prior to interview was not always possible or appropriate; (ii) self-classification of ethnicity was complex particularly for Black women and (iii) a larger number of Indian women were recruited in order to include women from Hindu and Sikh backgrounds (see supplementary information). We took the decision to recruit healthy women rather than cancer patients because we wanted to explore anticipated responses to a symptom without the potential retrospective bias associated with a cancer diagnosis 23 and because we felt community-based recruitment would be a more feasible way of reaching our target sample of BAME women within the time frame of the study.

Women were approached through community groups in the London boroughs of Brent, Barnet, Hounslow, Hillingdon, Newham and Lewisham. These boroughs were estimated to have at least 10% of their population from Caribbean or Indian backgrounds 19. Community groups were predominantly identified through online lists provided by councils, and their group leaders were contacted and asked to assist with study recruitment. In some cases, snowball sampling was used, whereby a community group leader suggested other groups that might like to be involved. Groups in Camden were also approached through a partnership-scheme with the university and an advertisement about the study was placed on Camden council website. Women were either approached by the group leader in person or *via* telephone (with details of interested people passed to the researcher) or responded to the researcher directly in person, by email or telephone after hearing about the study or seeing a poster.

### Procedure

Fifty-four interviews took place from June–October 2011. Most were facilitated by LM, but 13 interviews were carried out by an ethnically matched interviewer, eight in English, four in Punjabi and one in Hindi. Interviews took place at the participant's preferred location, at their home, community group venue or at the university and lasted around 40 min. A semi-structured interview schedule was used to guide the interviews. The structured part of the interview consisted of the following topics to be discussed: attitudes towards cancer in general, suggestions of what might be the symptoms of breast and cervical cancer, anticipated reactions to having such symptoms and the facilitators and barriers to help-seeking. Within each of these topics, open-ended questions were used, and the participant was encouraged to lead the discussion and raise points important to them. No symptoms were suggested by the interviewer. Interviews were transcribed verbatim by freelance transcribers and checked by LM. Where interviews were in a language other than English, they were transcribed into English and checked by a researcher fluent in that language. Prior to beginning the study, we sought advice on the study design and materials from a lay advisory panel of eight women aged 30–60 years from Indian (*n* = 4) and Black Caribbean backgrounds (*n* = 4). The study was approved by the UCL Research Ethics Committee.

### Analyses

Data were analysed using Thematic Analysis which involves the identification of themes within qualitative data 24. A ‘Framework’ approach was taken, using a ‘matrix-based analytic method that facilitates rigorous and transparent data management’ (p.220) 25. After familiarisation with the data (listening to recordings, reading and re-reading transcripts), a conceptual framework was developed by LM based predominantly on the data, but also taking symptom delay theory into consideration 1. LMcG familiarised herself with a subset of transcripts selected at random and checked the framework. Any discrepancies were resolved through discussion. We felt this cross-checking process would reduce subjectivity 26. The ‘framework’ feature in nvivo 9 (QSR International Pty Ltd., 2010) was used. Analysis involved close inspection of each theme in the framework, drawing on commonly mentioned attitudes and experiences.

## Results

The 54 women were from Indian–Hindu (*n* = 17), Indian–Sikh (*n* = 5), Indian–other religion (*n* = 2), Pakistani (*n* = 2), Bangladeshi (*n* = 2), Black Caribbean (*n* = 6), Black African (*n* = 2), Black British (*n* = 2), Black other (*n* = 2), White British (*n* = 11) and White other backgrounds (*n* = 3). Sample characteristics are shown in Table 1. For ease of description, the broad terms Asian, Black and White are used where appropriate. Women described a range of experiences with cancer and cancer symptoms. All women mentioned at least one person they knew (or knew of) who had cancer or cancer symptoms. Five women described personal experiences, one had breast cancer (P30), one had the BRCA2 gene (P7), two had sought help after finding a lump in their breast (P11, P23) and one had been called for further investigation after a mammogram (P53). Around half of the women described a close family member with cancer. Most other women had friends or colleagues who had cancer. In some cases, women had been closely involved throughout the person's illness (visiting, attending appointments and caring), whereas other experiences were more distant.

**Table 1:** Sample characteristics

	All women (*n* = 54)	White backgrounds (*n* = 14)	Black backgrounds (*n* = 12)	Asian backgrounds (*n* = 28)
Ethnicity				
White British	11	11	—	—
White other	3	3	—	—
Black British	2	—	2	—
Black Caribbean/mixed White & Black Caribbean	6	—	6	—
Black African	2	—	2	—
Black Other	2	—	2	—
Indian	24	—	—	24
Pakistani	2	—	—	2
Bangladeshi	2	—	—	2
Religion				
No religion	11	8	1	2
Christian	14	3	10	1
Hindu	17	0	0	17
Sikh	5	0	0	5
Other (inc. Muslim, Buddhist, Jewish and other)	7	3	1	3
Place of birth^a^				
Born in UK and parents born in UK	9	8	1	0
Born in UK and at least one parents born outside the UK	15	2	8	5
Neither self or parents born in UK	27	4	2	21
Age (years)				
25–34	7	3	1	3
35–44	15	5	5	5
45–54	23	3	5	15
55–64	9	3	1	5
Marital Status				
Single	20	5	9	6
Cohabiting	2	1	1	0
Married	26	4	2	20
Divorced/separated/widowed	6	4	0	3
Living Arrangement				
Rent from local authority	14	2	8	4
Rent from Private Landlord	5	2	1	2
Own Home	29	8	2	19
Other	6	2	1	3
Employment				
Employed full-time	21	7	3	11
Employed part-time	12	4	2	6
Unemployed	7	0	2	5
Full-time Homemaker	7	0	1	6
Other (retired/student/registered disabled)	7	3	4	0
Education				
No formal education or GCSE/O-level/CSE	13	0	4	9
A-Levels or equivalent	7	1	2	4
Degree or equivalent	27	10	5	12
Other	7	3	1	3

a Missing data for one woman.

We have structured the results under three broad headings consistent with the interview structure: *General beliefs about cancer, initial reactions to a symptom and facilitators and barriers to help-seeking*. Within each of these, a number of themes were identified from the data (Table 2) and these are discussed in the succeeding text with illustrative quotes. A number of themes appeared to be more prominent among BAME women, with different terminology used and different origins of beliefs described. These instances have been drawn out of the data. Direct quotes are followed by participant number, ethnicity and age.

**Table 2:** Summary of themes

General beliefs about cancer
Cancer fear
Discussing cancer
Lack of interest in cancer
Initial reactions to a symptom
Symptom appraisal
Self-management of symptoms
Social support
Facilitators and barriers to help-seeking
Cancer fear
Importance of early diagnosis
Health provider factors
System level factors
Competing priorities
Health values and conscientiousness

### General beliefs about cancer

#### Cancer fear

Cancer was seen as a feared disease by women from all ethnic groups. Fear was described as their ‘first thoughts’ or as an ‘automatic’ response to the word cancer. When asked why cancer was feared women discussed the perceived association between cancer and the inevitability of death; ‘if you get cancer, you know, your life, that's it, your life has ended, you'll never recover’ (P33, Indian, 45). Even though many women acknowledged that new treatments meant cancer was no longer always a ‘death sentence’, people who survived were described as the ‘lucky’ ones and the fact that death from cancer remains a possibility means there is still fear around the disease; ‘Even though I hear in the medical things that there is a cure… still I find a lot of people are dying’ (P28, Indian, 54). The fear of recurrence was also raised.

Some women described their fear of cancer as arising from the way cancer can ‘spread’ and ‘grow’; ‘Cancer is something that spreads, it's a disease that can take over. You know if you've got throat cancer it can go into your lung, it can go into your kidneys’ (P9, Black Caribbean, 37). The potential for cancer to ‘creep up on you’ and to affect anyone also contributed to fear around the disease; ‘It could be any one of us, and even a healthy lifestyle isn't enough to guarantee it’ (P18, White other, 41). Beliefs about cancer treatment contributed to fear of the disease, with treatments seen as ‘violent’, ‘horrific’ and ‘harsh’.

Women felt that their fear of cancer came both from the media and from their own experiences with the disease. Older women from BAME backgrounds described experiences where family members were diagnosed late. Many of these cases were in other countries (e.g. India and Pakistan), but there were also examples of parents or grandparents living in England who had cancer diagnosed at a late stage. Some of the younger women described experiences of friends or family members being successfully treated and felt this made them less afraid ‘I'm not scared of it now because my mum went through’ (P13, Indian, 38).

#### Discussing cancer

Some women from BAME backgrounds described how cancer was not traditionally discussed in their culture. Most women agreed that this was not the case among the younger generation brought up in the UK, and felt that they would discuss cancer openly with their children. The reasons for not discussing cancer seemed to vary. Asian women described a generic attitude to keeping health and illness topics private ‘When it comes to personal issues or talking about health … it's not appropriate to talk about’ (P17, Pakistani, 38). For women from Black African and Caribbean backgrounds, there were specific attitudes around cancer that meant it was not discussed; ‘Growing up in the West Indian community … cancer it was almost like it's a swear word’ (P26, Black Caribbean, 48).

#### Lack of interest in cancer

Several women said they had never taken much interest in cancer. Lack of interest was attributed to a range of reasons including young age, lack of experience with cancer making it less personally relevant and other priorities, ‘When you don't suffer from it or immediate family don't suffer, you tend to ignore it’ (P30, Indian, 54); ‘We get so busy with day-to-day things, we don't really pay much attention’ (P36, Indian, 46).

### Initial reactions to a symptom

#### Knowledge of symptoms

Most women mentioned a lump as a sign of breast cancer, but all other symptoms were only mentioned by a few women, for example, changes to breasts, nipple discharge and pain. The most common symptoms that women mentioned for cervical cancer were discharge and bleeding (in general, in-between periods or after sex). Other women mentioned painful sex, abdominal pain and itching. Several women acknowledged there are ‘not necessarily any great signs’. Some women mentioned ‘any kind of change’ as a symptom, whereas others openly said they did not know any symptoms of breast and/or cervical cancer.

#### Symptom appraisal

Women described how their reaction to a symptom was likely to depend on how serious they thought it was and whether it could be attributed to something else (e.g. menopause and getting older). Some women anticipated feeling uncertain about whether they would know if a symptom was serious, but others felt they would know instinctively if something was wrong because ‘you know your body’. Some said that they would not expect a symptom to be cancer because they did not feel at risk; either because they considered themselves to be healthy and take care of themselves (e.g. eat healthily and go for screening) or did not have a family history of cancer.

#### Self-management of symptoms

Most of the women said they would monitor their symptoms first (with time intervals from a few days to a month) to see if it would ‘just go away’. Women from Indian and White groups described how they would look up information on the Internet before seeking advice. Information seeking was not mentioned by women from Black backgrounds. A few women, predominantly although not exclusively from BAME backgrounds, discussed self-managing the symptom through prayer or lifestyle changes; ‘I'm a Christian so obviously for me I would pray, I would first of all take it to God’ (P12, Black African, 44); ‘(I'd) put myself on what I call a detox programme, lots of fruit and vegetables and flush my system’ (P47, Black Caribbean, 57). There was some general discussion about the use of alternative medicine and traditional remedies among a few of the women from Caribbean backgrounds. They felt that the older generation in particular would make attempts to ‘remedy it themselves’ using a ‘back home remedy’ or ‘own mother medication’ before seeking help.

#### Social support

Although many women said they would discuss symptoms with a friend or family member for reassurance or prompting before seeking medical advice, there were also some who said they would not talk to anyone about it. This was predominantly because they would not want to ‘worry them’. A few Asian women said they would not discuss the symptom because they would consider it to be private, and some of the older Indian women felt that concerns about symptoms of breast or cervical cancer would not be shared because they related to parts of the body that were not openly discussed in their culture.

### Facilitators and barriers to help-seeking

#### Cancer fear

Many women described fear of cancer as a motivator to seek help. Associations between cancer and death evoked a sense of urgency. As a result of this, seeking reassurance that the symptom was nothing to worry about was a key motivator to visit the GP; ‘I'd rather get it checked out and know rather than have the unknown and just sort of worry about it’ (P2, Indian, 28). This need for reassurance was raised more frequently by younger women, but was discussed across all ethnic groups. Conversely, not wanting to know was a barrier that women gave for delaying a visit to the GP. For some women, there was a conflict here; ‘I'd want to know, but not want to know’ (P10, Black other, 39). Fear of cancer was also described as a barrier to help-seeking by many of the women. Women felt the fear of being diagnosed with cancer and having to have treatment for cancer may contribute to delay; ‘You don't wanna be told you've got cancer, you'd rather, I think, imagine it's something else, or it's not a problem’ (P18, White other, 41).

#### The importance of early diagnosis

Most women mentioned the importance of early diagnosis of cancer for effective treatment as a trigger to seeking help; ‘I wouldn't leave anything at all because, you know the longer you leave it the less chance you've got of surviving’ (P21, White British, 44). For many the experience of a friend or family member who had been diagnosed at an early stage and survived or diagnosed late and died was a strong motivator to seek medical advice; ‘I just sort of look at my mum and what she went through and I don't want to go through the same thing … if it's too late’ (P13, Indian, 38). Personal-level previous experience of a symptom or an abnormal screening result was also considered to be a trigger to seeking help.

#### Healthcare provider factors

Women described their relationships with their GP as an important part of the decision to seek help. This was by far the dominant theme among all ethnic groups and all age groups. Some women described having a good relationship with their GP, trusting them and feeling that is what the GP is there for. However, others reported negative attitudes towards their GP as a barrier for delaying help-seeking. These women felt that they had no relationship with their GP, felt their GP was unapproachable or would be dismissive or did not trust their GP; ‘Doctors give off the vibe that oh it's nothing … you tend to think that they will brush you off’ (P11, Black African, 39). Past experiences in the medical setting played a role for some women. One woman described how being sick as a child influenced her help-seeking behaviour ‘I spent a lot of my childhood in hospital … when I get sick I will refuse to go and I will go at the last, last second’ (P9, Black Caribbean, 37). Two women (one Black Caribbean and one Pakistani woman) discussed past experiences of perceived racism as potential barriers to help-seeking. One Caribbean woman discussed how she felt about a White male GP examining a Black woman; ‘… a strange white man … you always remembering your history about all the horrible things that were done to females, Black females in those days and so it may just kind a run through your mind a bit’ (P47, Black Caribbean, 57). A few women discussed the nature of a symptom of breast or cervical cancer as relevant, describing the need to show these to the GP as potentially embarrassing; ‘Because of the region of the body that you're talking about, people would find it quite sort of personal and a bit embarrassed’ (P2, Indian, 28). Many of the Asian women described concerns about having to see a male GP.

#### System level factors

Several women from each ethnic group discussed practical barriers around getting an appointment that would delay help-seeking. This included difficulty getting an appointment, having to wait a long time for an appointment (especially to see a female GP) and having to tell the receptionist their symptoms. Difficulty getting time off work was also seen as a barrier. Some women also mentioned the limited time-slot allocated to appointments; ‘My whole issue is 8 min to undress, redress and be out of the door—in 8 min flat … I'd rather not bother going, cos I'd be so stressed out’ (P47, Black Caribbean, 57). Several women from Indian backgrounds mentioned how not speaking English well can make it difficult to communicate with the GP. Language was also felt to influence accessibility of the surgery, for example, by making it difficult to get buses and navigate maps.

#### Competing priorities

Competing priorities were frequently discussed by women from all ethnic groups as a barrier to delay help-seeking. Work and family life meant women felt they were often too busy to visit the GP. Some women described particular life events that would make them delay help-seeking, for example, family weddings, holidays and family illness. In particular, concern about the possible consequences of what might be found at a time in their lives they considered difficult would result in delay; ‘(It was a) difficult time for the whole family and I suppose if I had had a health worry at that point that wasn't terribly severe, I probably would have decided not to do anything about it … I had too much on my plate and I didn't want to cope with it’ (P46, White British, 52). Women from BAME backgrounds described how their family (husband and children) came first, and that a mentality to ‘put up with it’ was in their culture: ‘I think as a mother and a wife you soldier on and maybe you don't take things as seriously about yourself as you would as if it was a member of your family’ (P26, Black Caribbean, 48).

#### Health values and conscientiousness

Some women discussed how their help-seeking behaviour would be in line with the way they usually dealt with health issues; describing themselves as either the sort of person who puts things off or who gets things carried out. Younger women in particular tended to talk more pragmatically about their health, describing valuing health in general as a facilitator for seeking help; ‘For me health is really important and it's best to get it checked’ (P13, Indian, 38). Some women from BAME backgrounds also described how they valued their family life and would seek help promptly because of their children or grandchildren; ‘I value what we have in our family life and I wouldn't want anything to compromise that’ (P15, Indian, 43).

## Discussion

This study reports on qualitative interview data with women from a range of ethnic minority backgrounds adding to the literature on facilitators and barriers to symptom presentation which until now has been predominantly in White women. BAME groups tend to report longer intervals between noticing a cancer symptom and seeking help, yet minimal research has explored why this may be. This study aimed to identify facilitators and barriers to help-seeking that are distinct for women in these groups. A number of factors were raised by BAME women, for example, the use of alternative medicine or prayer to try and self-manage symptoms before considering medical help and the concept of women needing to ‘soldier on’ and prioritise the family over the need to seek-help for a symptom. Discussing symptoms with others was not always considered appropriate. Language problems and concerns about racism were also raised as two broader issues that influenced some women's attitudes to visit the GP in all circumstances. A number of our findings are consistent with a recent focus-group study of West African men and women in Luton 27.

Among all women regardless of ethnicity, the most common barrier to help-seeking was negative attitudes to the GP. The most common facilitator was the belief that early diagnosis was important for survival. Attributing a symptom to cancer, as opposed to something else (e.g. menopause and getting older) was also important. This is consistent with previous studies that have explored reported ‘reasons’ for delayed help-seeking in women who have been given a cancer diagnosis 8,9,17,18. Women's discussions of cancer fear and the factors that contribute to this were complex and included concerns about progression, recurrence and cancer treatments as well as death from the disease. These beliefs came largely from experience with cancer, and there were more stories of death from cancer recalled among BAME women than White women. Fear of the symptom being cancer was mentioned as both a facilitator and a barrier to help-seeking.

All women thought they would go to the GP if they found a symptom they thought might be cancer, but rather than an immediate response; women described a more considered process between noticing a symptom and seeking help. Some women felt they would make an appointment within days and others mentioned weeks or a couple of months, but none said they would delay for more than 3 months, the time interval that has been associated with later stage of cancer at diagnosis 2–4. This finding is consistent with other studies of healthy people, where most say they would seek help quickly 16. However, there are delays of more than 3 months in around a third of cancer cases 8. This is concerning because it suggests that either anticipated delay is not an accurate representation of actual delay behaviour or recruitment of healthy samples does not include those people who go on to delay (due to selection bias).

These findings have a number of implications. They indicate that models of help-seeking and delay in presentation that have been developed based on research with the White majority population may be largely transferable across different ethnic groups. Interventions designed to encourage help-seeking for a cancer symptom among BAME women should include similar information regarding the importance of early diagnosis, correctly attributing symptoms and addressing negative attitudes to the GP. However, campaigns may also benefit from addressing additional concerns about racism, the perceived benefits of self-management and the cultural beliefs about not discussing cancer and ‘soldiering on’. Channelling campaigns through a wide range of sources (TV, radio, print and community settings) will also increase the likelihood that public health messages are seen by those from all ethnicities.

Throughout the interviews, women from ethnic minority groups discussed generational differences in help-seeking in their cultures. Younger women emphasised their upbringing in the UK as an important aspect in their thinking about healthcare and cancer. Older women (i.e. those aged 50–65 years) who were born and brought up abroad described their integration in society as having an important influence on their attitudes, and felt that their parents' generation would approach the situation differently. These views emphasise the importance of maintaining an up-to-date research literature when exploring attitudes to cancer among minority women—the most recent studies in the UK were carried out 10 years ago.

There are a number of limitations to this study. The women participating in the interviews had not necessarily experienced any cancer symptoms and did not usually describe responses to actual symptoms, and we do not know if this hypothetical context reflects what would happen if symptoms were experienced. Much of the help-seeking literature has been based on inviting cancer patients to think back to their diagnosis pathway. There are limitations to retrospective studies, both in terms of memory issues, and because patients are discussing their past behaviour in the knowledge that the symptom was cancer. However, prospective studies are difficult because of the large samples needed to identify future cancer patients before they get symptoms. For example, in a population-representative survey in Britain, only 11% of men and women said they had experienced a symptom which they had worried might be cancer in the past 3 months, of whom 75% had seen a doctor to discuss this 28.

Despite being ethnically diverse, all women were recruited from London and many were well educated. The sample cannot be considered to be representative of the UK BAME population as a whole. Although population representativeness is not the aim of qualitative work, we cannot assume that the same themes would have arisen from a sample in another location or from other ethnic groups not included in this study, for example Indian–Muslim, Somali and Eastern Europeans. We also focused recruitment on women aged 25–64 years, and therefore we cannot be sure that there would not be different themes among older women. The age range we included meant most women were acculturated, spoke English, were employed in the UK and mixed with other cultures. There is some suggestion that in qualitative studies the ethnicity of the interviewer can influence the findings. We, therefore, commissioned ethnically matched interviewers to carry out a selection of the interviews. However, there did not appear to be any differences between the issues discussed in the English non-matched, English matched and non-English matched interviews, consistent with the previous findings 29. This could be because of the age range of women included in this sample. Finally, this study was with women only focussing on breast and cervical cancer and research with BAME men about other cancers may have drawn different conclusions.

To our knowledge, at the time of data collection, this was the first published study to explore facilitators and barriers to help-seeking for a cancer symptom among women from a diverse range of ethnic backgrounds in the UK. The findings suggest that some facilitators and barriers to help-seeking among women from predominantly Indian and Black Caribbean backgrounds do not differ greatly from White women. Whether the symptoms were attributed to cancer, beliefs in the importance of detecting cancer early and attitudes to the GP were the dominant themes across all groups, however, ethnicity does seem to influence these attitudes. For example, beliefs about the importance of early detection can be influenced by discussing cancer openly, which does not happen in some cultures and perceived racism and language-barriers can contribute to negative attitudes towards the GP. Encouraging open discussion about cancer among minority communities could help raise awareness about the importance of early detection and thus promote help-seeking. Further research is needed to design and test interventions for decreasing delay in help-seeking among BAME women.

## Note

Sufficient inclusion of women from all ethnic minority groups would have resulted in a need for almost 100 interviews and this was beyond the scope of the study.
