# Supplementary material for: Facilitators and barriers to help-seeking for breast and cervical cancer symptoms: a qualitative study with an ethnically diverse sample in London
Source: Psychooncology. 2013 Dec 19;23(7):749–57. doi: 10.1002/pon.3464 (PMC4282580; doi:10.1002/pon.3464)
Supplement: Supplementary file 1 [file pon0023-0749-SD1.pdf]

Supplementary Information: Recruitment context

Reasons for deviation from the original purposive sampling frame

After approaching the first few community groups it emerged that directing recruitment specifically to certain subgroups was not always considered acceptable. In particular, community groups aimed at women from black backgrounds did not distinguish between Caribbean and African and did not feel it was acceptable for us to do so. This was also the case in some of the groups for Asian women. As a result a number of women who were not in the original target groups indicated interest in the study. The inclusion criteria were therefore adjusted, although the focus on Indian and Caribbean women remained. Self-classified ethnicity among women from black backgrounds was complex. Despite describing an upbringing in the Caribbean when asked to complete the questionnaire indicating their ethnic group several of the women selected black other, sometimes writing themselves an additional box for ‘black British’. After meeting the recruitment target for Indian women we realised that all were from Hindu backgrounds. We therefore decided to continue recruiting Indian women, changing the focus to the Sikh community and therefore the overall number of interviews with Indian women was more than the other groups.

General comment

The study plan was first devised at the end of 2009. We planned to recruit through community groups in areas of London with high proportions of women from Caribbean and Indian ethnic backgrounds. Following drastic cuts in public funding which began in 2010, 50% of community groups in London were forced to close. Remaining groups cut staff and many remaining staff had to allocate most of their time to writing funding applications. As a result engaging community groups in the present study was difficult. In Summer 2011, during the recruitment period, a young black man was killed by police officers in North London, raising questions about racism and authority. Following this event, women from black backgrounds that were approached in community groups responded negatively to research focusing on ethnicity. As a result recruitment of women from black backgrounds was difficult.
